# Implementation of circulating tumor DNA (ctDNA) testing in precision oncology: A four-year experience from a tertiary cancer center in India

**DOI:** 10.1016/j.jlb.2025.100319

**Published:** 2025-07-26

**Authors:** Pradnya Joshi, Prachi Gogte, Prachi Pawar, Mamta Gurav, Ramya Iyer, Shambhavi Singh, Sonam Hatkar, Ujwal Shetty, Aruna Nair, Mansi Mulay, Snehal Jaiswar, Trupti Pai, Gauri Deshpande, Nupur Karnik, Prarthna Shah, Aditi Arora, Archita Juneja, Sangeeta Desai, Omshree Shetty, Tanuja Shet

**Affiliations:** a Molecular Pathology Laboratory, Department of Pathology, Homi Bhabha National Institute, Tata Memorial Hospital, Mumbai, India; b Department of Pathology, Homi Bhabha National Institute, Tata Memorial Hospital, Mumbai, India; c Department of Medical and Precision Oncology, Sir HN Reliance Foundation Hospital and Research Centre, Mumbai, 400004, Maharashtra, India

**Keywords:** Liquid biopsy, Circulating tumor DNA, ctDNA, Next-generation sequencing, Targeted panel testing, Non-invasive testing

## Abstract

**Introduction:**

Liquid biopsy testing has emerged as a pivotal tool in molecular characterization of solid malignancies. Circulating tumor DNA (ctDNA) analysis is quintessential in precision oncology for early detection, disease monitoring, prognosis, and theranostic purposes. This study summarizes ctDNA analysis performed on patients with advanced or metastatic solid tumors at tertiary cancer centers in India (2021–2024).

**Methods:**

ctDNA was isolated following standard pre-analytical protocols and quality control measures. Sequencing was performed using the Oncomine Precision Assay on Thermo Fisher platform and Custom Solid Tumor Panel (SOPHiA Genetics) on Illumina platforms. Variant annotation and clinical interpretation were performed as per ACMG and AMP Guidelines.

**Results:**

Out of 236 ctDNA samples, majority were lung malignancies (47 %), gastric cancers (43 %), head & neck cancers (2 %), other malignancies (8 %). A total of 250 clinically relevant genomic alterations were reported. On Illumina, 19.8 % of variants were classified as Tier I, 18.3 % Tier II, and 11.7 % as Tier III. Thermofisher platform identified Tier I alterations in 33 %, Tier II in 54 %, and Tier III in 11 % cases. In the GI cohort, *TP53* was most frequently mutated (51 %), followed by *KRAS* (25 %), *BRAF* (13 %), *PIK3CA* (13 %), and *CHEK2* (9 %). Among lung cancer patients, *EGFR* mutations (44 %), followed by *TP53* (43 %), *CDKN2A* (9 %), *PIK3CA* (9 %), and *BRAF* (6 %).Tissue–liquid biopsy concordance was observed in 36 of 96 cases for which baseline tissue NGS data was available. The mutational landscape derived from this cohort was compared with the MSKCC dataset for Asian and Western populations. The comparison revealed high concordance, clinical relevance, and reliability of liquid biopsy-based genomic profiling in diverse oncologic settings.

**Conclusion:**

Study underscores substantial increase in the adoption of liquid biopsy and the real-world utility of ctDNA-based NGS testing in solid tumors contributing to improved patient care management.

## 1 Introduction

Cancer remains one of the leading causes of morbidity and mortality worldwide, despite recent advancements the burden is disproportionately borne by low- and middle-income countries. There is an increasing need for precision-based strategies in early detection, diagnosis, disease monitoring, and treatment selection. Traditionally, tissue biopsy has been the gold standard for obtaining tumor-specific molecular information. However, it is an invasive approach and hence scenarios wherein repeat or serial testing is needed, obtaining tissue gets cumbersome [1,2]. Additionally in cases with multiple metastatic sites to capture spatial and temporal heterogeneity of tumors and to capture subclonal progression, liquid biopsy has an upper edge. Liquid biopsy approaches involving the analysis of tumor-derived materials such as circulating tumor DNA (ctDNA), circulating tumor cells (CTCs), exosomes, and other nucleic acids present in body fluids are emerging as a reliable surrogate for invasive tumor tissue based diagnostic testing [3]. Among these, ctDNA has gained considerable attention for its potential to reflect the dynamic molecular landscape of tumors in real-time. Blood remains the most commonly utilized sample source, although other fluids—including urine, pleural and ascitic effusions, cerebrospinal fluid (CSF), and saliva—are also being explored with promising results [4].

The primary advantages of liquid biopsy include its minimally invasive nature, the ability to perform serial sampling, rapid turnaround time, and its capacity to capture a broader representation of tumor heterogeneity by sampling systemic disease [5,6]. Liquid biopsy has been extensively used in solid malignancies to uncover actionable mutations, monitor therapeutic response or identify resistance mechanisms and outcomes.

Study from Massachusetts General Hospital Cancer Center and Harvard Medical School has demonstrated that ctDNA can help identify risk levels in patients with metastatic colorectal cancer, supporting the use of liquid biopsies in deciding the treatments and follow-up strategies leading to improved outcomes [7]. Similarly, a study from East Asia of 1608 patients demonstrated that ctDNA-based Next generation sequencing (NGS) in non-small cell lung cancer (NSCLC) provided timely and useful clinical information that can be included in the standard diagnostic process for NSCLC [8].

However, despite these advantages, liquid biopsy presents several limitations. The detection of ctDNA is often constrained by its low abundance, particularly in early-stage cancers or tumors with minimal shedding [9]. Furthermore, the variability in ctDNA release across different tumor sites may affect its reliability in representing the full tumor burden [10]. Currently, there is a lack of consensus on standardized workflows, standard operating procedures (SOPs) for critical steps such as sample collection, ctDNA extraction, library preparation and robust evidence supporting its role in clinical decision-making is evolving. Pre-analytical variables significantly impact the quality and detection of actionable biomarkers, thereby affecting downstream analysis. Moreover, liquid biopsy provides information limited to specific molecular alterations and may not fully reflect the biological complexity and heterogeneity of the disease. Enhancing the sensitivity and specificity of detection platforms remains essential, particularly for identifying low-abundance ctDNA, which is vital for early detection and minimal residual disease monitoring [[11], [12], [13]].

Notably, the European Society for Medical Oncology (ESMO) does not recommend the use of ctDNA-based assays for minimal residual disease (MRD) detection outside clinical trials, citing insufficient evidence for their impact on altering treatment outcomes [14].

In India, the adoption of ctDNA-based NGS testing in clinical practice has been limited, with relatively few large-scale datasets available from real-world oncology settings. At our institute, which is a high-volume tertiary referral cancer center, liquid biopsy using ctDNA has been routinely offered for select clinical indications since 2020. This study presents a retrospective evaluation of liquid biopsy NGS in treatment-naïve or advanced/metastatic solid tumors conducted over four years (2021–2024). We aim to assess the effectiveness of liquid biopsy versus standard tissue testing and analyze genomic alterations, identify co-occurring mutations, and concordance between tissue-based testing, thereby contributing to the growing body of evidence for integrating liquid biopsy into routine oncologic care in the Indian subset population.

## 2 Materials and methods

### 2.1 Study population

This study is a retrospective audit of the routine diagnostic cases which were received at the Division of Molecular Pathology, Tata Memorial Hospital (TMH), Mumbai, India between 2021 and 2024. The study included all the cases of liquid biopsy which were subjected to targeted NGS panel testing. The liquid biopsy samples (n = 236) were obtained from patients diagnosed with advanced-stage or metastatic disease. The patient population majorly included a subset of solid tumors including lung, gastrointestinal (GI), head and neck (H & N) and breast cancer cases.

### 2.2 Cell-free DNA (cfDNA) extraction

Peripheral blood samples were collected in cfDNA-stabilizing tubes (e.g., Streck cfDNA BCT®) to preserve nucleic acid integrity and prevent cellular lysis. All samples were handled in accordance with ISLB (International society of liquid biopsy) best practice recommendations [15]. Samples were transported at ambient temperature and were received in the laboratory within 24 h of collection to ensure optimal cfDNA integrity.

Upon receipt, samples were promptly processed using a two-step centrifugation protocol. The first spin was performed at 1600×*g* for 10 min at 4 °C to separate plasma, followed by a second centrifugation at 16,000×*g* for 10 min at 4 °C to remove any remaining cellular debris. The plasma was carefully aliquoted (avoiding the buffy coat) and stored at −80 °C until further use, with care taken to avoid multiple freeze–thaw cycles.

cfDNA was extracted from 2 to 4 mL of plasma using the COBAS® cfDNA Sample Preparation Kit (Roche Diagnostics, Germany), following the manufacturer's instructions. Quantification of cfDNA was performed using the Qubit dsDNA High Sensitivity Assay (Thermo Fisher Scientific), and cfDNA fragment size distribution was assessed using the Agilent Tape Station 4200 with the Cell-Free DNA Screen Tape assay (Agilent Technologies, USA). cfDNA fraction was calculated by selecting the range of 100 to 200bp in the size selection settings to ensure the maximum enrichment of the possible ctDNA, only samples demonstrating a cfDNA fraction >10 %, based on internal laboratory cutoffs, were selected for downstream NGS analysis.

### 2.3 Targeted Next generation sequencing

Samples were sequenced using two different platforms: one based on Manual hybrid capture-based chemistry and the other using Automated amplicon-based chemistry.

Hybrid capture NGS panel using manual protocol: This panel comprises of 55 genes for somatic variant detection, 24 genes for somatic copy number alterations (SCNAs), and 6 loci for microsatellite instability (MSI) analysis. Sequencing was carried out on the Illumina NextSeq 2000 platform.

Amplicon-based automated NGS Panel: Oncomine Precision Assay (OPA) covers 50 hotspot genes and 16 CNVs.

#### 2.3.1 Manual library preparation using hybrid capture chemistry

Manual library preparation was carried out using the SOPHiA™ Solid Tumor Solution (STS) Plus kit on the SOPHiA DDM® platform (SOPHiA Genetics, Switzerland), utilizing hybrid capture-based chemistry. cfDNA samples underwent end-repair and adapter ligation, followed by individual sample library amplification under cycling conditions as per manufacturers protocol [ Supplementary Data (S1)]. Each library was then subjected to quality assessment and equimolar pooling (200 ng per sample; 8–12 samples per pool). Hybridization was performed overnight at 65 °C for 16 h, followed by streptavidin bead capture and stringent post-hybridization washes. Post-capture amplification and purification steps were subsequently performed to generate the final libraries, which were quantified using Qubit and Tape Station systems. A final concentration of 450 pM of pooled libraries was loaded onto the Illumina NextSeq platform for sequencing (details provided in Supplementary Data S1).

Sequencing metrics included an average variant depth of coverage around 10,000 × and a variant allele frequency (VAF) threshold of >0.1 %, ensuring high sensitivity for detecting low-frequency tumor-derived variants. Data were analyzed using the SOPHiA DDM bioinformatics pipeline, and only samples meeting quality control criteria (coverage uniformity >80 % and Q_30_ >90 %) were retained for downstream analysis. Variant annotation was performed using multiple databases, including ClinVar, COSMIC, dbSNP, 1000 Genomes, OncoKB, Franklin Genomics, and gnomeAD. Variants were interpreted according to ACMG (American College of Medical Genetics and Genomics), AMP (Association for Molecular Pathology) guidelines and reported using HGVS (Human Genome Variation Society) nomenclature.

#### 2.3.2 Automated amplicon-based library preparation

Automated targeted sequencing was performed using the Genexus System (Thermo Fisher Scientific, USA), a fully integrated, walk-away platform. DNA was extracted, quantified using the Qubit and loaded into the Oncomine Precision Assay (OPA) cartridges as per the manufacturer's instructions. The OPA panel covers 50 hotspot genes and 16 CNVs. Library preparation, sequencing, and primary analysis were completed on the Genexus platform using amplicon-based chemistry.

Sequencing output was uploaded to the Ion Reporter software for secondary and tertiary analysis. Quality control parameters were as follows (average coverage >10,000 × , VAF >0.1 %, coverage uniformity >80 %, Q_20_ >90 %) were applied. Annotated variants from the Ion Reporter pipeline were similarly reviewed using established clinical databases and reported according to ACMG, AMP, and reported using HGVS nomenclature.

## 3 Statistical analysis

Clinical and demographic characteristics of the patient cohort, including age, gender, cancer subtype, and disease stage, were summarized using descriptive statistics. Median and range were reported for continuous variables, while frequencies and percentages were used for categorical variables.

To assess the similarity in mutational patterns, Pearson correlation coefficients (r) were calculated between the Current study cohort and the dataset retrieved from cBioportal.

## 4 Results

### 4.1 Clinical characteristics of the cohort

A total of 236 patients were subjected to blood-based NGS testing of ctDNA. Of these, one sample did not meet the sequencing quality matrix criteria and was excluded from further analysis. Thus, 235 patients were included in the final dataset. The median age was 57 years. A majority of the patients presented with advanced-stage disease (n = 188; 80 %) and had documented metastases (n = 195; 83 %), Male: Female ratio of the study population was 1.46:1.

### 4.2 Actionable mutational landscape

**Fig. 1:**
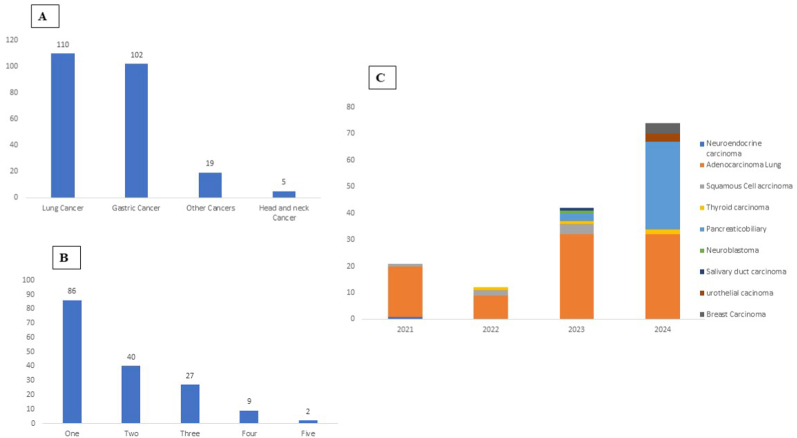
**Summary of case distribution by organ site, number of genetic alterations, and annual liquid biopsy requests.** (A) Distribution of analyzed cases according to organ site. (B) Distribution of cases based on the number of detected genetic alterations (ranging from 1 to 5). (C) Annual trend in the number of liquid biopsy requests received during the study period.

The study cohort comprised patients diagnosed with GI cancers (n = 102; 43.2 %), lung cancers (n = 110; 46.6 %), H & N cancers (n = 5; 2.1 %), and other malignancies (n = 19; 8.1 %), which included urological, gynecological, breast, and neurological cancers (Fig. 1A). Assay performance metrics and detailed demographic information are provided in Table 1.

**Table 1:** Sequencing platform performance matrix and demographic detail.

Parameters	Illumina	Thermofisher
	Next Seq and Miseq	Genexus
Total no of Cases (n = 236)	136	100
Genomic alterations not detected	68 (50 %)	2 (2 %)
Uninterpretable Cases	1 (0.72 %)	0
Avg variant Depth	7861X	21881X
Avg VAF detected	15.89	9.03
Min VAF	0.03	0.1
Max VAF	86.5	95
Tier I	27 cases (19.8 %); 36 alterations	33 cases (33 %); 51 alterations
Tier II	25 cases (18.3 %); 54 alterations	54 cases (54 %); 100 alterations
Tier III	16 cases (11.7 %); 38 alterations	11 cases (11 %); 14 alterations
Male: Female	1.46:1	

The analysis of the trend of liquid biopsy testing requested across the different types of malignancies demonstrates a consistent rise in the number and diversity of malignancy cases from 2021 to 2024. Notably, while adenocarcinoma lung remained the most frequent indication, there was a sharp increase in pancreaticobiliary and breast carcinoma cases in 2024, reflecting evolving clinical adoption across tumor types (Fig. 1C).

Targeted profiling revealed a total of 250 clinically relevant genomic alterations, classified according to ASCO/AMP/ACMG guidelines. Among cases analyzed using the Illumina platform, 19.8 % were classified as Tier I, 18.3 % as Tier II, and 11.7 % as Tier III variants. In contrast, the Thermo Fisher platform identified Tier I alterations in 33 % of cases, Tier II in 54 %, and Tier III in just 11 % of cases.

Among these, 241 were single-nucleotide variants (SNVs), 51 were insertions/deletions (indels/frameshifts), and 8 were copy number variations (CNVs). A single-gene alteration was observed in 86 patients, with *TP53* being the most frequently altered gene. Multiple gene alterations (≥2 genes) were detected in 78 patients (Fig. 1B).

### 4.3 Genomic landscape of GI cancers

The GI cancer cohort comprised 102 patients with diverse histological subtypes, comprising of adenocarcinoma (n = 89; 86 %), gastrointestinal stromal tumors (GISTs) (n = 4; 3.9 %), Cholangiocarcinoma (n = 3; 2.9 %), poorly differentiated carcinoma and signet ring cell adenocarcinoma (n = 2; 2 % each), and mucinous as well as neuroendocrine tumors (n = 1; 1 %) each.

**Fig. 2:**
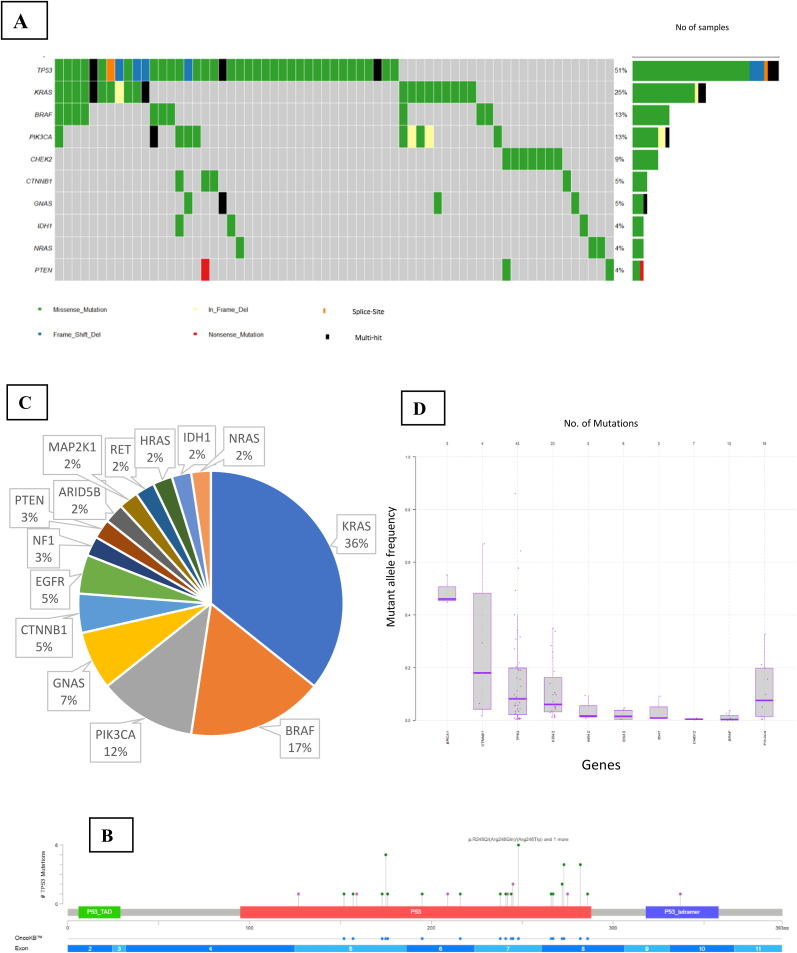
**Mutational landscape of gastric cancer.** (A) Oncoplot showing the top 10 most frequently altered genes in Gastric cancer. (B) Lollipop plot depicting the mutation spectrum of *TP53* gene in Gastric cancer. (C) Co-occurrence profile of *TP53* mutations with other gene alterations in Gastric cancer. (D) Distribution of mutant allele frequencies of alterations detected in Gastric malignancies.

Among these 102 patients, 143 genomic alterations were identified in 79 individuals. The most frequently altered gene was *TP53*, detected in (n = 40; 51 %) of patients, followed by *KRAS* (n = 20; 25 %), *BRAF* (n = 10; 13 %), *PIK3CA* (n = 10; 13 %), and *CHEK2* (n = 7; 9 %). The distribution of the top 10 altered genes in the GI cohort is visualized in the oncoplot in Fig. 2A. Analysis of the Mutant allele frequency of the top altered genes in the GI malignancies showed a wider range across the samples which included the following genes: *TP53, KRAS*, and *PIK3CA* genes, depicted in Fig. 2D.

Since most patients in the cohort had GI adenocarcinomas, we analyzed the mutational landscape within this subgroup. The top five actionable alterations in GI adenocarcinomas was reported in *TP53* (49 %), followed by *KRAS* (29 %), *PIK3CA* (13 %), *BRAF* (10 %), and *EGFR* (3 %) genes. Other histological subtypes, including GISTs, poorly differentiated carcinomas, and cholangiocarcinomas each reported alterations in two cases. In addition, signet ring cell adenocarcinoma, neuroendocrine tumors, mucinous carcinoma, and epithelioid hemangioendothelioma patients showed alterations in one case each. Supplementary Data S2 provides an Oncoplot representing the mutational profile of GI adenocarcinomas, along with a table summarizing the mutations observed in other histological subtypes.

Consistent with prior clinical trials conducted at Tata Memorial Hospital [16], *TP53* mutations are associated with poor survival in gastric adenocarcinomas. Of the 46 *TP53* mutations observed in the current GI cohort, 44 were missense mutations and 2 were indels, spanning exons 4 to 10. The distribution and type of *TP53* mutations are shown in Fig. 2B.

Co-occurrence analysis revealed that *KRAS* (36 %), *BRAF* (17 %), and *PIK3CA* (12 %) mutations were the most frequent partners of *TP53* mutations in this cohort (Fig. 2C).

### 4.4 Genomic landscape of lung malignancies

During the study period, the majority of liquid biopsy tests were performed for lung cancer patients (n = 110), predominantly adenocarcinoma cases (n = 98; 89 %), followed by squamous cell carcinoma (n = 7,6.4 %), poorly differentiated carcinoma (n = 4; 3.7 %), and neuroendocrine tumors (n = 1,0.9 %).

**Fig. 3:**
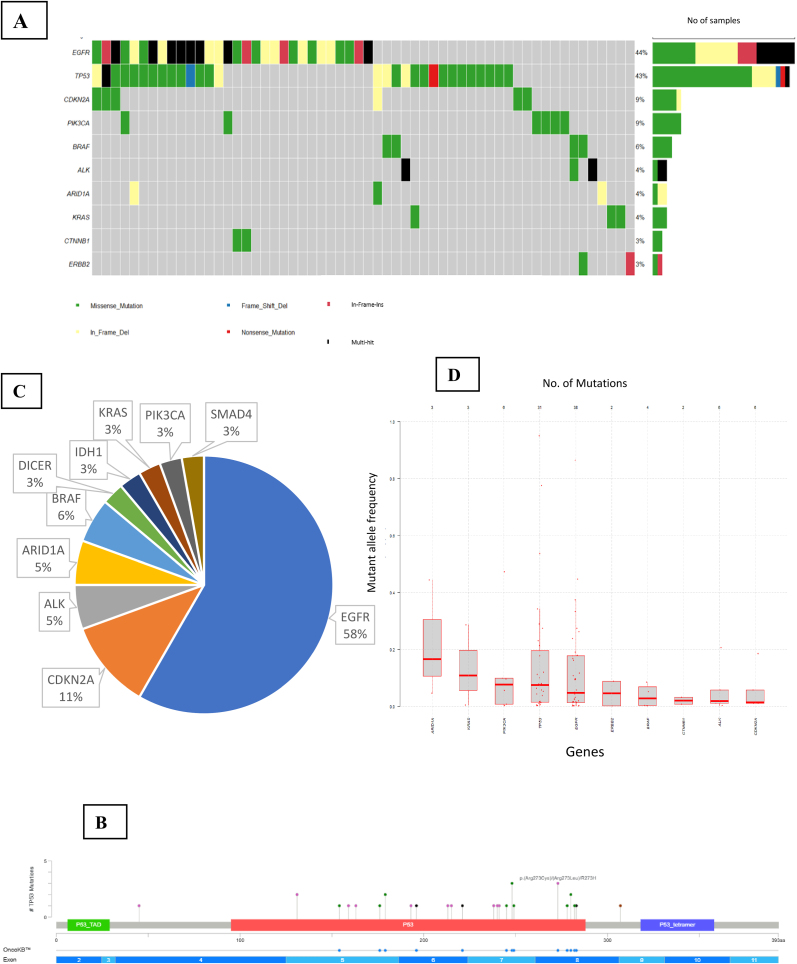
**Mutational landscape of lung cancer.** (A) Oncoplot representing the top 10 most frequently altered genes in Lung cancer. (B) Lollipop plot illustrating the spectrum of *TP53* gene mutations identified in Lung cancer cases. (C) Co-occurrence profile of *TP53* mutations with other gene alterations in Lung cancer. (D) Distribution of mutant allele frequencies of alterations detected in Lung malignancies.

A total of 123 genomic alterations were detected in 68 patients. *EGFR* mutations were the most frequent (n = 30; 44 %), followed closely by *TP53* (n = 29; 43 %), with additional alterations in *CDKN2A* (n = 6; 9 %), *PIK3CA* (n = 6; 9 %), and *BRAF* (n = 4; 6 %). The oncoplot in Fig. 3A summarizes the top 10 most commonly altered genes in this lung cancer cohort. Analysis of the Mutant allele frequency of the top altered genes in the lung malignancies showed a wider range across the samples in *TP53* and *EGFR* genes, depicted in Fig. 3D.

As with GI cancers, the majority of lung cancer patients in our cohort were diagnosed with adenocarcinoma. Therefore, we focused our mutational analysis on this subgroup. The top five actionable alterations in lung adenocarcinomas were reported in *EGFR* (43 %), *ALK* (5 %), *BRAF* (5 %), *KRAS* (5 %), and *ROS1* (2 %) genes. Among the non-adenocarcinoma subtypes, squamous cell carcinoma exhibited detectable alterations in five cases, and poorly differentiated carcinomas showed mutations in three cases. No alterations were identified in cases diagnosed as neuroendocrine tumors. Supplementary Data S3 presents an Oncoplot summarizing the mutational landscape of lung adenocarcinomas, along with a detailed table outlining mutations observed across other histological subtypes.

*TP53* mutations, known to be predictive of poor outcomes in lung cancer, were examined in greater detail. These mutations were detected in 32 patients comprising 34 unique alterations—30 missense mutations and 4 indels—spanning exons 4 to 8. Fig. 3B displays the *TP53* mutation map for this cohort.

Among patients with *TP53* mutations, the most frequent co-occurring alterations were in *EGFR* (58 %) and *CDKN2A* (11 %), as presented in Fig. 3C.

### 4.5 Genomic landscape of other malignancies

The cohort also included cases from other malignancies, comprising gynecological cancers (n = 6), pediatric solid tumors (n = 1), urological cancers (n = 4), breast cancers (n = 5), neurological cancer (n = 1), and cancers of unknown primary origin (n = 2).

**Fig. 4:**
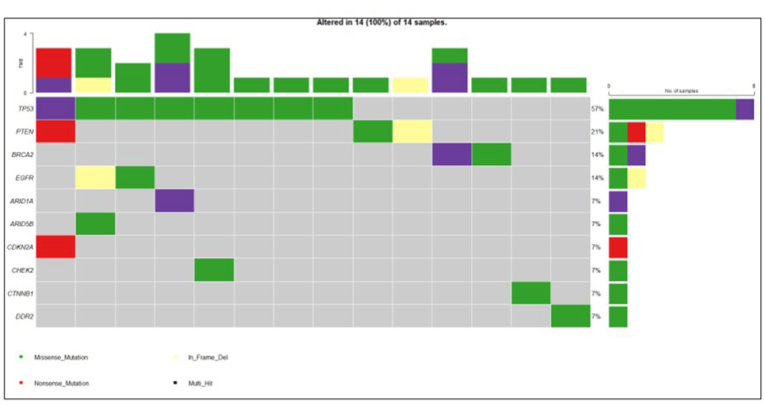
**Mutational landscape across other malignancies.** Oncoplot displaying the top 10 most frequently altered genes identified in malignancies other than gastric and lung cancer.

Analysis of the mutational landscape of these cases revealed *TP53* as the most frequently altered gene (n = 8; 57 %) followed by *PTEN* (n = 3; 21 %), *EGFR* (n = 2; 14 %), *BRCA2* (2; 14 %). Fig. 4 represents the Oncoplot of the top 10 altered genes in this diverse cohort.

### 4.6 Concordance between tissue and liquid biopsy

Baseline clinical data were retrievable for 161 patients. Of these, 96 patients had tissue biopsy samples on which the same targeted NGS panel was performed. The data was available at the time of initial diagnosis. Concordance in genomic alterations between baseline tissue and liquid biopsy NGS was observed in 36 cases. Among discrepant cases, 9 cases were liquid biopsy positive/tissue negative, and 14 cases, baseline mutations detected in tissue NGS were not observed in the ctDNA.

### 4.7 Mutation frequency comparison with MSKCC (Memorial Sloan Kettering Cancer Center) data

We retrieved ctDNA mutation data from the MSKCC dataset [17] from cBioportal for both Asian and Western cohorts and compared the mutation frequencies with our current dataset. As the TMH cohort predominantly included lung and gastric cancer cases, only these subtypes were considered for comparative analysis.

**Fig. 5:**
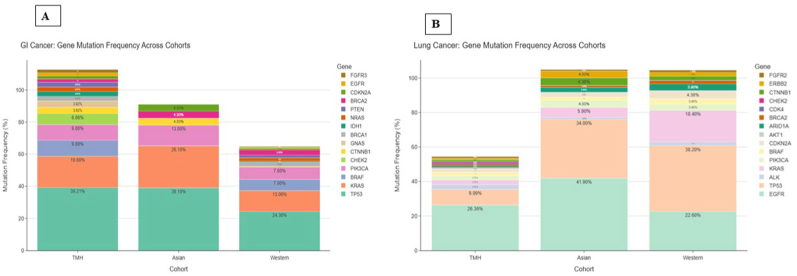
**Comparative gene mutation frequency in gastric and lung cancers: Asian and Western populations versus current study data.** (A) Comparative analysis of gene mutation frequencies in gastric cancer across Asian and Western populations relative to the current dataset. (B) Comparative analysis of gene mutation frequencies in lung cancer across Asian and Western populations relative to the current dataset.

For gastric cancer, the mutational profile showed a correlation coefficient of r = 0.93 and r = 0.970 respectively with both Asian and Western populations. Similarly, lung cancer cases exhibited a correlation of r = 0.647 with the Western cohort and r = 0.914 with the Asian cohort (Fig. 5).

**Fig. 6:**
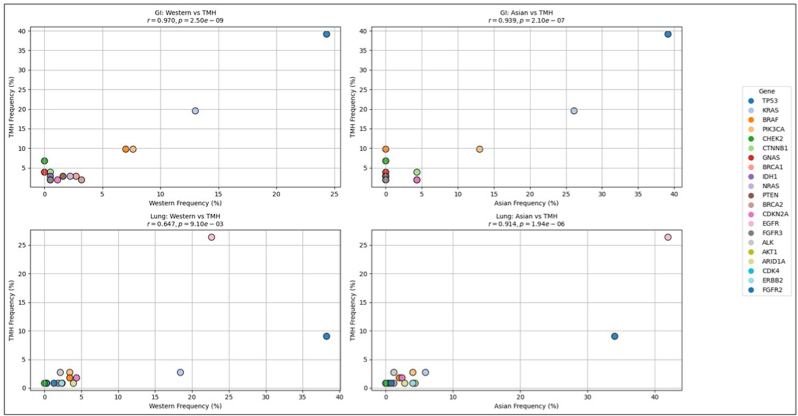
**Correlation of gene mutation profiles between current data and global datasets.** Correlation analysis comparing the gene mutation frequencies observed in the current dataset with those reported in Asian and Western populations of publicly available MSKCC (Memorial Sloan Kettering Cancer Center) dataset.

**Fig. 7:**
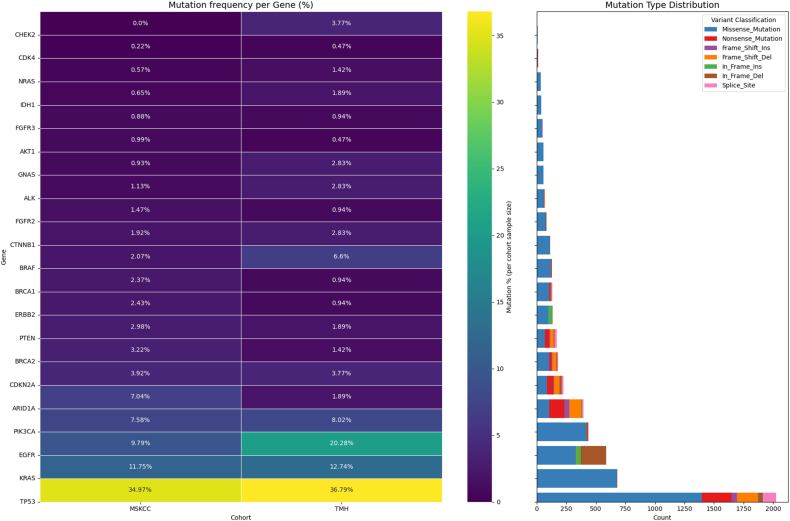
**Comparative distribution of mutation types and frequencies: current dataset vs. MSKCC.** Parallel comparison of mutation types and frequencies between the present study data and publicly available MSKCC dataset.

When comparing the overall mutation frequency of lung and gastric cancer cases between the TMH and MSKCC cohorts, a broadly similar mutational landscape was observed. However, the TMH cohort showed relatively higher frequencies of mutations in genes such as *BRAF*, *CHEK2*, *EGFR*, *CTNNB1*, *ALK*, and *GNAS* (Fig. 6) (see Fig. 7).

Although the TMH cohort (n = 212) is considerably smaller than the MSKCC dataset (n = 5567), this comparative analysis provides valuable insight into the distribution of common targetable alterations across different populations. These observations highlight the need for further validation in larger, more diverse cohorts to better understand population-specific genomic patterns and their clinical relevance.

## 5 Discussion

There are several clinical applications for ctDNA in pan-cancer setting and it is most advanced in lung, breast and GI malignancy [18,19]. There is growing evidence in clinical applications of this approach at different time points including early detection, prognosis, monitoring of treatment response, detection of residual disease and relapse in early stages, detection of progression and emergence of resistance mutations in advanced/metastatic cancers and as a biomarker to guide treatment selection [20]. As an increasing number of actionable mutations are uncovered for various malignancies, a practical approach to reveal complete genotype is needed so that more patients can receive a tailored targeted therapy. Tissue for NGS has its own limitations such as low tumor content, poor fixation, tissue necrosis, depletion of the tissue as well as acquisition of tissue biopsy from patients. ctDNA-based NGS testing helps to overcome these challenges and hence has gained popularity. ctDNA-based NGS is utilized in clinical trials particularly in lung and GI cancer [[21], [22], [23]]. In addition to discovering biomarkers, most liquid NGS studies have rapid turnaround times of molecular testing and initiating treatment.

Although ctDNA-based NGS has gained momentum in clinical oncology and is being increasingly incorporated into routine practice, several practical and systemic challenges remain. These include analytical complexities, high cost of testing, and the requirement for dedicated infrastructure to ensure optimal sample handling, transportation, and timely processing. While efforts to formalize guidelines for ctDNA testing are underway, their adoption into real-world workflows is still at an early stage.

Our observations align with the broader literature, which remains heterogeneous across tumor types and regions. Ethnic and geographic variations significantly influence the genomic landscape and studies from Southeast Asia/Asia Pacific remain underrepresented in pan-cancer liquid biopsy analyses. This gap limits our understanding of region-specific molecular alterations and their therapeutic implications. Nonetheless, owing to variations in genomic characteristics and socioeconomic status between Asian and Western populations, the application of liquid biopsy varies across Asia-Pacific regions [24].

The availability of proposed frameworks and consensus guidelines [[25], [26], [27]] for interpreting the clinical utility of genomic alterations is a positive step, but more data from diverse populations are needed to validate and refine these approaches.

Supporting this, the Korean Lung Liquid Versus Invasive Biopsy Program demonstrated the added value of ctDNA-based NGS when used alongside tissue genotyping in advanced NSCLC. Their findings reinforce that ctDNA can complement traditional testing by identifying additional actionable alterations, particularly when tissue is limited or inaccessible. This underscores the importance of context-specific evaluation of liquid biopsy utility in improving molecular stratification and clinical outcomes across various malignancies [28].

The present study offers a real-world perspective on the evolving role of liquid biopsy-based NGS in routine oncology practice, representing one of the first such evaluations from a tertiary cancer center in India. The findings in this cohort were also compared with the MSKCC data set and were consistent with the findings. The comparison of molecular markers observed in the GI and Lung cancer cohorts were concordant, however since its a single centre data the numbers were relatively smaller to achieve statistical significance. This study highlights the evolving adoption of liquid biopsy-based NGS in routine oncology practice, particularly in GI and thoracic cancers. Initially used as a substitute when tissue was unavailable or inadequate, liquid biopsy has transitioned into a complementary tool used alongside tissue testing. Increasingly, clinicians are requesting liquid biopsy at baseline and for longitudinal monitoring, reflecting a shift in its perceived clinical value.

The data show a clear trend toward integrating liquid biopsy for treatment monitoring, MRD detection, and even considering a "liquid-first" approach in select advanced cases where rapid molecular insights are needed. This changing pattern underscores growing confidence among oncologists in the reliability and clinical relevance of ctDNA testing, driven by efficient assay performance, faster turnaround times, and cost-effective diagnostic workflows. Our experience with both automated and hybrid-capture–based workflows demonstrates that well-optimized ctDNA testing can be achieved at an approximate cost of ₹20,000 (∼$233 USD/€199) per sample. These estimates include the cost of nucleic acid extraction, quality control, library preparation, sequencing reagents, data analysis on the cloud based bioinformatics as well as other necessary consumables.

This study has certain limitations that may impact the broader applicability of its findings. As a single-center analysis using a small targeted NGS panel, the scope of detectable genomic alterations was restricted, and the lack of RNA fusion detection may have resulted in missed clinically relevant events. Variability in ctDNA fraction among patients, influenced by tumor burden and disease site, could have further affected the sensitivity of mutation detection, especially in low-shedding tumors.

While the clinical utility of liquid biopsy-based NGS is expanding, challenges remain in interpreting low allele frequency variants, especially in early-stage disease, and in the absence of standardized clinical pathways to guide treatment decisions based on ctDNA findings. Cost-effectiveness remains a significant concern for large-scale adoption, particularly in low- and middle-income settings. In our experience, high testing costs and logistical hurdles in maintaining serial blood collections limited longitudinal monitoring, despite willingness from patients and clinicians. These challenges highlight the need for scalable, affordable solutions to enable wider and more consistent integration of liquid biopsy into routine cancer care. To highlight the Asian perspective, Korea and Japan located in East Asia have improved economic resources supporting reimbursement of NGS, while it is not the same case thus accessibility to molecular tests and liquid biopsy is still a challenge in the Indian population [[29], [30], [31]].

## 6 Conclusion

This study demonstrates the real-world utility of liquid biopsy-based NGS for detecting clinically relevant genomic alterations in patients with lung and GI cancers. Despite limitations in scale and infrastructure, the successful implementation of this noninvasive approach highlights its potential to complement tissue-based testing and guide treatment decisions. As the landscape of precision oncology continues to evolve, our findings support the integration of ctDNA profiling into routine clinical workflows and underscore the need for cost-effective, scalable solutions to expand access, particularly in resource-constrained settings. Broader validation in larger, diverse populations will be key to realizing the full clinical impact of liquid biopsy in cancer care.

## Authors Contribution

OS conceptualized and designed the entire study. PJ, PB, MG, RI analyzed the data. PP performed the bioinformatics analysis. RI, US, SH, SJ, AN, PJ, MM performed experiments. OS, PJ wrote the manuscript. TP, NK, GD, PS, AA, AJ, PB, RI, MG, SD, TS critically reviewed and edited the manuscript. SS edited the manuscript and referencing. SD and TS provided approved the final draft and the overall study. All authors have read and approved the final manuscript.

## Statement of ethics

The authors declare the study was exempted from ethical committee review in accordance with the author's institution as this is a retrospective study. De-identified retrospective data was reviewed. Informed consent was waived.

## Date availability statement

Data that support the findings of this study are available on request from the corresponding author. The data are not publicly available due to privacy or ethical restrictions.

## Funding sources

None.

## Declaration of competing interest

The authors declare that they have no known competing financial interests or personal relationships that could have appeared to influence the work reported in this paper.

## Appendix A Supplementary data

Multimedia component1 **S1:** Detailed NGS protocol for hybridization capture based workflow along with QC matrix. Multimedia component1

Multimedia component2 **S2:** Oncoplot and classification of detected variants in lung and gastrointestinal (GI) malignancies based on AMP and ESMO recommendations, presented in detail according to tutor histology. Multimedia component2
