# Supplementary material for: Implementation of circulating tumor DNA (ctDNA) testing in precision oncology: A four-year experience from a tertiary cancer center in India
Source: J Liq Biopsy. 2025 Jul 26;9:100319. doi: 10.1016/j.jlb.2025.100319 (PMC12356032; doi:10.1016/j.jlb.2025.100319)
Supplement: Multimedia component1 — S1: Detailed NGS protocol for hybridization capture based workflow along with QC matrix. [file mmc1.docx]

**SOPHIA CSTS PANEL PROTOCOL FOR LIBRARY PREPARATION**

**Fragmentation & end repair**.


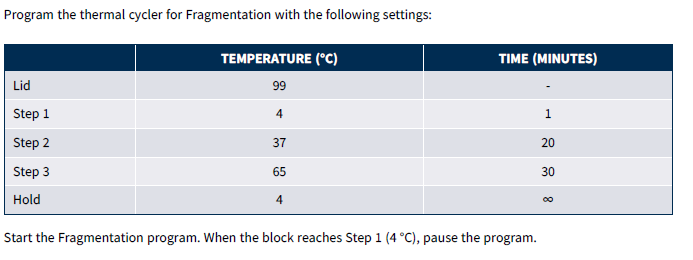


**Ligation**

Incubate the plate/tubes in the thermal cycler at 20°C for 15 minutes (open lid).

**Post-ligation clean up (as per protocol)**

**Library amplification**

(N=15cycles)


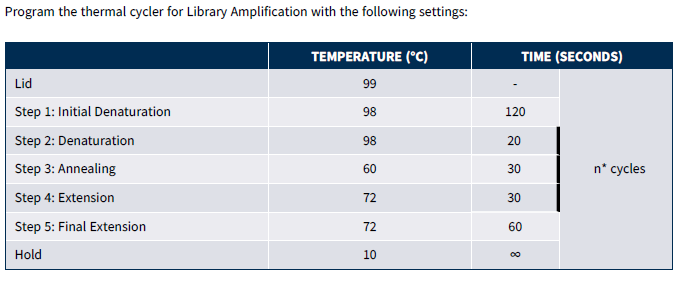


**Post amplification clean up (as per protocol)**

**Individual library quantification and quality control**

1. Libraries were Quantified using the Qubit
2. Quality control the libraries were analyzed by capillary electrophoresis. Library DNA fragments should have a size distribution between 200 bp and 800 bp.

**Library pooling**

Post Quantifications samples (8-12) samples were pooled in the single pool followed by Lyophilization

Lyophilized pools were reconstituted in the hybridization buffer and an appropriate amount of probe, and the pools were incubated at 65°C overnight in a thermal cycler for 16 hrs

**Post hybridization capture**

Capturing of the libraries with Streptavidin beads binding to the hybridized products, followed by post-hybridization washes

**Post capture amplification**

**(n=10cyccles)**


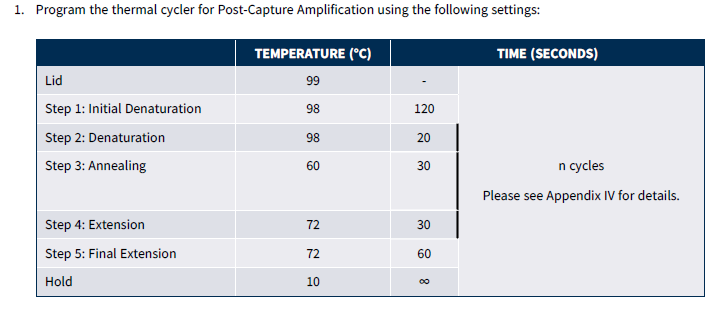


**Post capture amplification cleanup(as per protocol)**

**Final library quantification and quality control**

1. Libraries were Quantified using the Qubit
2. Quality control the libraries were analyzed by capillary electrophoresis. Library DNA fragments should have a size distribution between 200 bp and 800 bp.

**Loading on to the Nexseq machines**

Library Concentration: 450pM/500pM/550pM (According to the type of flow cell)
