# Supplementary material for: Implementation of circulating tumor DNA (ctDNA) testing in precision oncology: A four-year experience from a tertiary cancer center in India
Source: J Liq Biopsy. 2025 Jul 26;9:100319. doi: 10.1016/j.jlb.2025.100319 (PMC12356032; doi:10.1016/j.jlb.2025.100319)
Supplement: Multimedia component2 — S2: Oncoplot and classification of detected variants in lung and gastrointestinal (GI) malignancies based on AMP and ESMO recommendations, presented in detail according to tutor histology. [file mmc2.docx]

**Actionable Genes in Lung Adenocarcinoma**


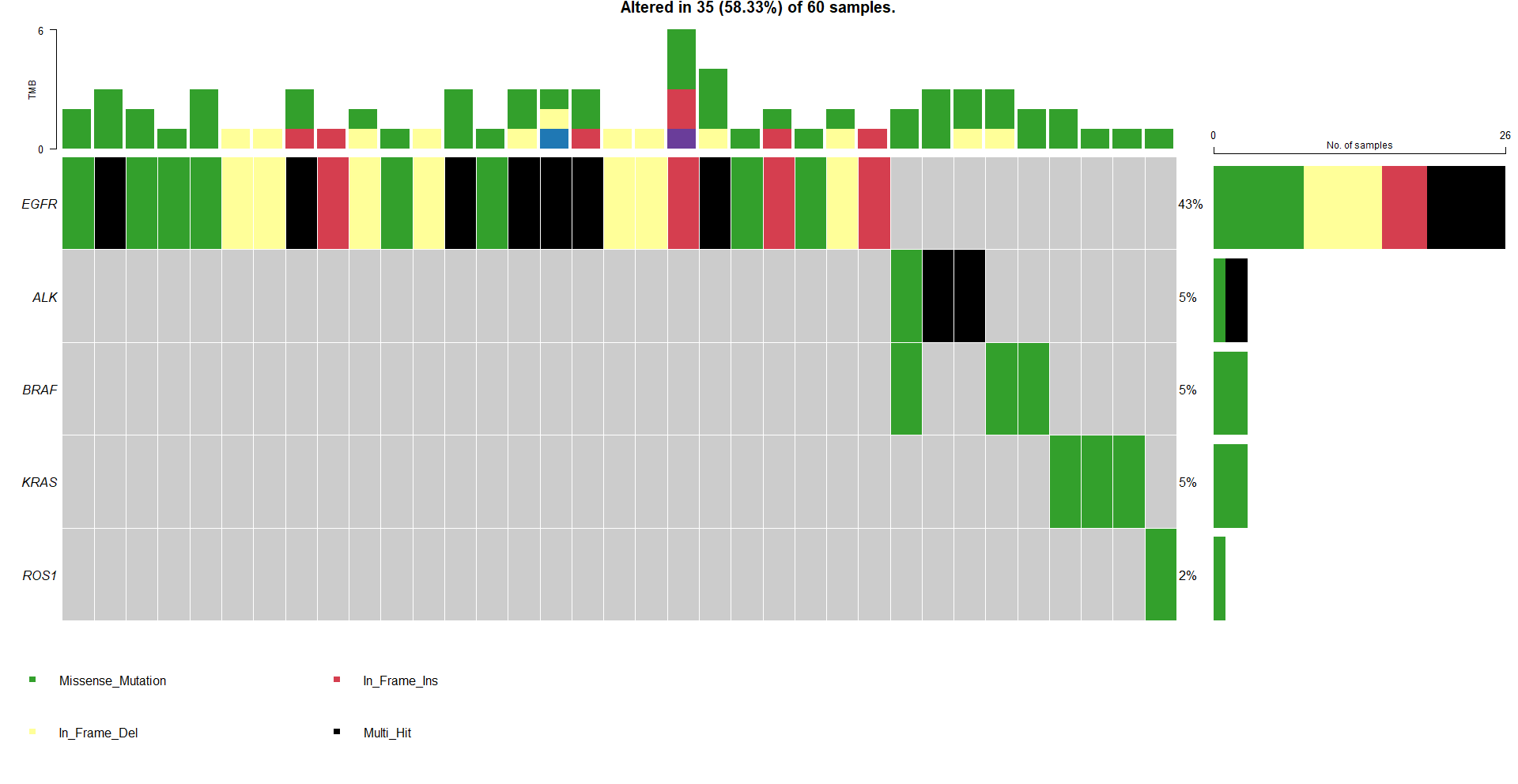

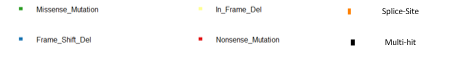


No of samples

**Actionability scores based on AMP and ESMO recommendations in Lung Adenocarcinoma**

| **Histology** | **Gene** | **Protein Change** | **AMP/ACMG Score** | **ESCAT Score** |
| --- | --- | --- | --- | --- |
| **Lung Adenocarcinoma**  **(99 Cases)** | *MYOD1* | p.N2025S | III | X |
|  | *EGFR* | p.L858R | I | I/II |
|  | *EGFR* | p.E746_A750del | I | I/II |
|  | *CDKN2A* | p.E69G | I | I/II |
|  | *BRAF* | p.V600E | I | I/II |
|  | *TP53* | p.R273C | II | III/IVA |
|  | *TP53* | p.C238S | II | III/IVA |
|  | *PIK3CA* | p.R524K | III | X |
|  | *EGFR* | p.V769M | II | III/IVA |
|  | *TP53* | p.S215I | II | III/IVA |
|  | *CDK4* | p.R61K | III | X |
|  | *TP53* | p.R273L | II | III/IVA |
|  | *EGFR* | p.T790M | I | I/II |
|  | *TP53* | p.A159P | II | III/IVA |
|  | *CDKN2A* | p.L65P | II | III/IVA |
|  | *EGFR* | p.L747_P753delinsS | I | I/II |
|  | *TP53* | p.N131I | II | III/IVA |
|  | *TP53* | p.sS241* | III | X |
|  | *EGFR* | p.E746_S752delinsV | I | I/II |
|  | *TP53* | p.H193R | II | III/IVA |
|  | *DICER1* | p.Q1726Gfs*8 | II | III/IVA |
|  | *TP53* | p.G244_G245delinsVR | III | X |
|  | *CDKN2A* | p.A30V | III | X |
|  | *SMAD4* | p.W524C | III | X |
|  | *TP53* | p.S240G | II | III/IVA |
|  | *TP53* | p.L45Yfs*2 | III | X |
|  | *FOXL2* | p.M220Tfs*86 | II | III/IVA |
|  | *GNAQ* | p.N246_R247ins22 | III | X |
|  | *EGFR* | p.E709A | I | I/II |
|  | *TP53* | p.R213Q | II | III/IVA |
|  | *EGFR* | p.S752_I759del | I | I/II |
|  | *TP53* | p.R248G | II | III/IVA |
|  | *ALK* | p.I1171N | I | I/II |
|  | *TP53* | p.? | III | X |
|  | *ALK* | p.V1180L | I | I/II |
|  | *MET* | p.P1091S | III | X |
|  | *EGFR* | p.C797G | I | I/II |
|  | *SMAD4* | p.? | III | X |
|  | *ARID1A* | p.S506_Q555dup | III | X |
|  | *STK11* | p.E98* | II | III/IVA |
|  | *PIK3CA* | p.I718V | III | X |
|  | *FGFR2* | p.P253Afs*94 | III | X |
|  | *BRCA2* | p.A3029V | III | X |
|  | *PTEN* | p.E23V | III | X |
|  | *TP53* | p.R306_A307insX[66] | III | X |
|  | *ARID1A* | p.M967V | III | X |
|  | *CDKN2A* | p.A36Rfs*17 | II | III/IVA |
|  | *TP53* | p.H179Y | II | III/IVA |
|  | *ARID1A* | p.S265del | III | X |
|  | *TP53* | p.R280G | II | III/IVA |
|  | *EGFR* | p.L747_A750delinsS | I | I/II |
|  | *EGFR* | p.A750P | I | I/II |
|  | *PIK3CA* | p.E542K | II | III/IVA |
|  | *KRAS* | p.G12A | I | I/II |
|  | *EGFR* | p.L747_S752delinsQ | I | I/II |
|  | *TP53* | p.G154V | II | III/IVA |
|  | *TP53* | p.R283AfsTer62 | II | III/IVA |
|  | *TP53* | p.P278L | II | III/IVA |
|  | *TP53* | p.R280K | II | III/IVA |
|  | *EGFR* | p.E746_S752delinsI | I | I/II |
|  | *EGFR* | p.S768N | II | III/IVA |
|  | *TP53* | p.C176S | II | III/IVA |
|  | *EGFR* | p.V774M | II | III/IVA |
|  | *EGFR* | p.G724S | I | I/II |
|  | *TP53* | p.R196* | II | III/IVA |
|  | *KRAS* | p.G13D | I | I/II |
|  | *KRAS* | p.G12D | I | I/II |
|  | *TP53* | p.R282W | II | III/IVA |
|  | *ALK* | p.A1280V | III | X |
|  | *BRAF* | p.D594N | II | III/IVA |
|  | *ERBB2* | p.R896H | III | X |
|  | *KIT* | p.N822K | II | III/IVA |
|  | *CHEK2* | p.R523H | III | X |
|  | *ROS1* | p.G2032R | I | I/II |
|  | *TP53* | p.R248W | II | III/IVA |
|  | *AKT1* | p.E17K | II | III/IVA |
|  | *TP53* | p.E221* | II | III/IVA |
|  | *CTNNB1* | p.S37C | II | III/IVA |
|  | *TP53* | p.R273H | II | III/IVA |
|  | *PIK3CA* | p.E545K | I | I/II |
|  | *IDH1* | p.R132C | II | III/IVA |
|  | *CTNNB1* | p.G34V | II | III/IVA |
|  | *ERBB2* | p.Y772_A775dup | I | I/II |
|  | *GNAS* | p.R201C | II | III/IVA |
|  | *TP53* | p.R249S | I | I/II |
|  | *ALK* | p.G1202R | I | I/II |
|  | *ALK* | p.I1171S | I | I/II |
|  | *ALK* | p.L1196M | I | I/II |

**Actionability scores based on AMP and ESMO recommendations in other histology of Lung Cancer**

| **Histology** | **Gene** | **Protein Change** | **AMP/ACMG Score** | **ESCAT Score** |
| --- | --- | --- | --- | --- |
| **Poorly Differentiated Adenocarcinoma**  **(3 Cases)** | *EGFR* | p.L858R | I | I/II |
|  | *TP53* | p.G244_G245delinsVR | III | X |
|  | *CDKN2A* | p.A30V | III | X |
|  | *SMAD4* | p.W524C | III | X |
|  | *EGFR* | p.T790M | I | I/II |
|  | *EGFR* | p.C797G | I | I/II |
|  | *SMAD4* | p.? | III | X |
|  | *BRAF* | p.G466E | I | I/II |
|  | *ERBB2* | p.R896H | III | X |
|  | *KIT* | p.N822K | II | III/IVA |
| **Squamous cell carcinoma (5 Cases)** | *TP53* | p.Y163C | II | III/IVA |
|  | *EGFR* | p.E746_A750del | I | I/II |
|  | *TP53* | p.L45Yfs*2 | III | X |
|  | *GNA11* | p.? | III | X |
|  | *EGFR* | p.L747_T751del | I | I/II |
|  | *ARID1A* | p.S265del | III | X |
|  | *TP53* | p.R280G | II | III/IVA |
|  | *PIK3CA* | p.E542K | II | III/IVA |

**Actionable Genes in GI Adenocarcinoma**

**
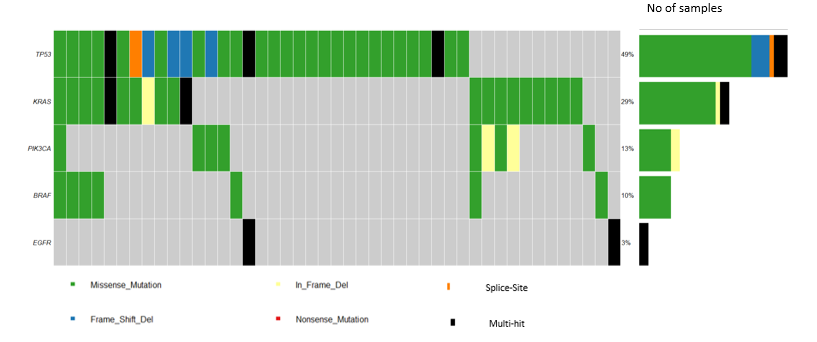
**

**Actionability scores based on AMP and ESMO recommendations in GI Adenocarcinoma**

| Histology | Genes | Protein Change | AMP/ACMG Score | ESCAT Score |
| --- | --- | --- | --- | --- |
| GI Adenocarcinoma  (89 cases) | *CTNNB1* | p.D32N | II | III/IVA |
|  | *PIK3CA* | p.H1047R | II | III/IVA |
|  | *CDKN2A* | p.L65P | II | III/IVA |
|  | *KRAS* | p.G12D | I | I/II |
|  | *BRCA1* | p.G1801D | III | X |
|  | *TP53* | p.C242Afs*5 | II | III/IVA |
|  | *BRCA1* | p.D1546H | III | X |
|  | *GNA11* | p.? | III | X |
|  | *BRCA1* | p.F1036Lfs*12 | II | III/IVA |
|  | *KRAS* | p.G12A | I | I/II |
|  | *TP53* | p.C275Y | II | III/IVA |
|  | *NF1* | p.S2694P | III | X |
|  | *TP53* | p.R248Q | II | III/IVA |
|  | *KRAS* | p.G12F | II | III/IVA |
|  | *MAP2K1* | p.F53V | II | III/IVA |
|  | *BRAF* | p.V600E | I | I/II |
|  | *CTNNB1* | p.D32H | II | III/IVA |
|  | *CHEK2* | p.R523H | III | X |
|  | *RET* | p.R912W | III | X |
|  | *CTNNB1* | p.G34R | II | III/IVA |
|  | *TP53* | p.R282W | II | III/IVA |
|  | *PTEN* | p.G127E | I | I/II |
|  | *NRAS* | p.G12S | II | III/IVA |
|  | *TP53* | p.G245AfsTer2 | II | III/IVA |
|  | *TP53* | p.R175H | II | III/IVA |
|  | *CDKN2A* | p.R58* | II | III/IVA |
|  | *TP53* | p.V173L | II | III/IVA |
|  | *MAP2K1* | p.L215P | II | III/IVA |
|  | *IDH1* | p.R132C | II | III/IVA |
|  | *PIK3CA* | p.E81K | III | X |
|  | *KRAS* | p.G12V | II | III/IVA |
|  | *RAF1* | p.S257L | II | III/IVA |
|  | *PTEN* | p.R173H | II | III/IVA |
|  | *GNAS* | p.R201H | II | III/IVA |
|  | *ESR1* | p.V392I | II | III/IVA |
|  | *BRAF* | p.G469E | II | III/IVA |
|  | *KRAS* | p.G12C | I | I/II |
|  | *EGFR* | p.G465R | II | III/IVA |
|  | *KRAS* | p.A146T | I | I/II |
|  | *TP53* | p.V216M | II | III/IVA |
|  | *TP53* | p.G266V | II | III/IVA |
|  | *BRAF* | p.A598V | II | III/IVA |
|  | *KRAS* | p.Q61H | I | I/II |
|  | *TP53* | p.V157F | II | III/IVA |
|  | *BRAF* | p.K601E | II | III/IVA |
|  | *IDH1* | p.R132H | II | III/IVA |
|  | *NRAS* | p.Q61R | I | I/II |
|  | *EGFR* | p.V769M | I | I/II |
|  | *FGFR3* | p.R399C | III | X |
|  | *KRAS* | p.G12C | I | I/II |
|  | *FGFR2* | p.C382G | II | III/IVA |

**Actionability scores based on AMP and ESMO recommendations in other histology of GI Cancer**

| **Histology** | **Gene** | **Protein change** | **AMP/ACMG Score** | **ESCAT Score** |
| --- | --- | --- | --- | --- |
| **Signet Ring Cell Adenocarcinoma (1 Case)** | *TP53* | p.G244D | II | III/IVA |
| **GIST**  **Gastro Intestinal Stromal Tumor (GIST)**  **(2cases)** | *FGFR3* | p.R248C | I | I/II |
|  | *CHEK2* | p.R523H | III | X |
| **Neuroendocrine tumour**  **Neuroendocrine Tumor**  **(1 Case)** | *CTNNB1* | p. G34E | II | III/IVA |
|  | *PTEN* | p. Y225* | II | III/IVA |
|  | *ARID1A* | p. S934R | III | X |
|  | *TP53* | p. A159P | II | III/IVA |
| **Mucinous adenocarcinoma**  **(1 Case)** | *BRAF* | p. V600M | III | X |
|  | *BRCA2* | p. Q1138* | II | III/IVA |
|  | *RET* | p. T636_R694dup | III |  |
| **Poorly differentiated carcinoma (2 Cases))** | *BRAF* | p.E586K | II | III/IVA |
|  | *TP53* | p.C176Y | II | III/IVA |
|  | *BRAF* | p.A598T | III | X |
|  | *PIK3CA* | p.E545D | II | III/IVA |
|  | *PIK3CA* | p.H1047R | II | III/IVA |
|  | *TP53* | p.R273C | II | III/IVA |
| **Cholangiocarcinoma**  **(2 Cases)** | *TP53* | p.R267P | II | III/IVA |
|  | *TP53* | p.R248W | II | III/IVA |
| **Epithelioid Hemangioendothelioma**  **(1 Case)** | *TP53* | p.V272M | II | III/IVA |
